# Prognostic role of immune cells in hepatocellular carcinoma

**DOI:** 10.17179/excli2020-1455

**Published:** 2020-06-03

**Authors:** Meenakshi Sachdeva, Sunil K Arora

**Affiliations:** 1 Department of Translational & Regenerative Medicine, Post Graduate Institute of Medical Education and Research (PGIMER), Chandigarh, India; 2 Department of Immunopathology & Department of Translational & Regenerative Medicine, Post Graduate Institute of Medical Education and Research (PGIMER), Chandigarh, India

**Keywords:** immune cells, hepatocellular carcinoma, prognosis, immune checkpoint molecules

## Abstract

Hepatocellular carcinoma (HCC), with rising incidence rates, is the most commonly occurring malignancy of the liver that exerts a heavy disease burden particularly in developing countries. A dynamic cross-talk between immune cells and malignant cells in tumor microenvironment governs the hepatocarcinogenesis. Monitoring immune contexture as prognostic markers is quite relevant and essential to evaluate clinical outcomes and to envisage response to therapy. In this review, we present an overview of the prognostic value of various tumor infiltrating immune cells and the continually evolving immune checkpoints as novel biomarkers during HCC. Tumor infiltration by immune cells such as T cells, NK cells and dendritic cells is linked with improved prognosis and favorable outcome, while the intra-tumoral presence of regulatory T cells (Tregs) or myeloid derived suppressor cells (MDSCs) on the other hand is associated with poor clinical outcome. In addition to these, the overexpression of negative regulatory molecules on tumor cells also provides inhibitory signals to T cells and is associated with poor prognosis. The limitation of a single marker can be overcome by advanced prognostication models and algorithms that evaluate multiple prognostic factors and ultimately aid the clinician in improving the disease free and overall survival of HCC patients.

## Introduction

Hepatocellular carcinoma (HCC) is the most occurring cancer of the liver (75-85 %) that engenders morbidity particularly in developing countries (Bray et al., 2018[5]). Globally HCC exerts a heavy disease burden with more than 800,000 newly diagnosed cases annually accounting for close to 700,000 deaths each year. The primary risk factors include viral infections (Hepatitis B and C), liver cirrhosis and non-alcoholic liver disease. Among environmental factors, predisposition to toxins and lifestyle factors like alcohol consumption, smoking, diet, metabolic profile and obesity are also reported to be associated with pathogenesis of HCC (Rawla et al., 2018[53]). Chronic inflammation causing sustained damage to the liver leads to cirrhosis of liver, which most commonly culminates into HCC. 

The incidence rates of HCC have almost tripled in the past four decades, while death rates have almost doubled. Men are about three times more susceptible than females with liver cancer being the fifth most common cancer for men and seventh for women. As per American Cancer Society estimates (https://cancerstatisticscenter.cancer.org/), the expected number of new cases is 42,820 while the estimated deaths due to HCC would be 30,160 in the year 2020. The incidence rate continues to be 8.3 %, while the death rate stays at 6.6 %. Traditionally, HCC diagnosis is based on cytological or histological examination, however, with recent technological developments, patients with cirrhotic liver can be diagnosed with CT or MRI scans without the need of a biopsy. Several classification systems are available for staging HCC patients and also for prognostication, most widely accepted being the Barcelona clinic liver cancer stage classification system. Depending on the stage of tumor and extent of liver damage, treatment strategy is adopted using a multidisciplinary approach for the best clinical outcome (Yang et al., 2019[74]). The options include curative resection followed by liver transplantation, tumor ablation using radio frequency and microwaves. For non resectable HCC cases, trans-arterial embolization and radiotherapy are employed. Pharmaceutical drugs like Sorafenib are clinically approved as a first line treatment in patients with advanced stage. 

The biology of any cancer is linked to alterations in numerous signaling pathways as in case of liver tissue, which is associated with hepatocarcinogenesis. The tumor environment, that is continually exposed to numerous antigens, has a substantial role in evading the host defense mechanisms against the tumor. The status and roles of various immune effector cells including factors released by them play a critical role in either promoting or impeding the cancer progression (Sachdeva et al., 2015[54]). In HCC, tumor tissue is generally enriched with regulatory T cells and cytokines released by these cells suppress the effector T cell functions thereby playing an important role in anti-tumor immunity. Besides, an increased expression of various inhibitory molecules such as PD-L1, TIM3 etc. on tumor cells, is shown to induce T cell anergy and apoptosis, which contributes to immune evasion. 

Despite the intensive research efforts undertaken for diagnosis and treatment strategies to manage HCC, limited attention is paid to estimate prognosis, that is the probability of a particular outcome in a span of time. Prognostic markers can be measured and monitored to predict the progress of a disease or response to a therapeutic regimen. These markers can be used to estimate chances of recovery from a medical condition or the probability of recurrence of a disease. The success of a therapy depends on envisaging a patient's response to therapy and overall survival of patients. Therefore, identification of potential prognostic factors will be instrumental in better management of disease and decision making. With advancements in molecular techniques like next generation sequencing (NGS), numerous key genes have been found to be useful as prognostic markers (Jiang et al., 2020[31]). Since the frequency of tumor infiltrating lymphocytes (TILs) correlate with disease outcomes, their role as prognosis relevant markers is increasingly being considered. However, mere evaluation of numbers may not be sufficient, hence the contribution of their functional status should also be taken into account for a pragmatic prognostic outcome. In addition, integration of approaches such as assigning immune score to assess different types of immune cells rather than a single cell type could predict patient's survival more efficiently (Wei et al., 2020[69]). Here, we review in detail different types of TIL subsets in HCC prognosis and tools to assess the potential utility of these markers. Table 1(Tab. 1) (References in Table 1: Cai et al., 2006[8]; Chew et al., 2010[14]; Chew et al., 2012[13]; Dong et al., 2016[17]; Garnelo et al., 2017[24]; Gu et al., 2011[25]; Huang et al., 2012[29]; Ido et al., 1994[30]; Li et al., 2017[39]; Sun et al., 2015[60]; Wang et al., 2016[68]; Xiao et al., 2013[72]; Xu et al., 2019[73]; Yao et al., 2017[76]; Yin et al., 2003[78]; Yu et al., 2020[79]; Zhang et al., 2009[82]; Zhang et al., 2016[83]; Zhang et al., 2019[85]) summarizes all these studies evaluating the prognostic significance of various immune cells in HCC.

## Immune Cells Associated with Good Prognosis in HCC

**Figure 1:**
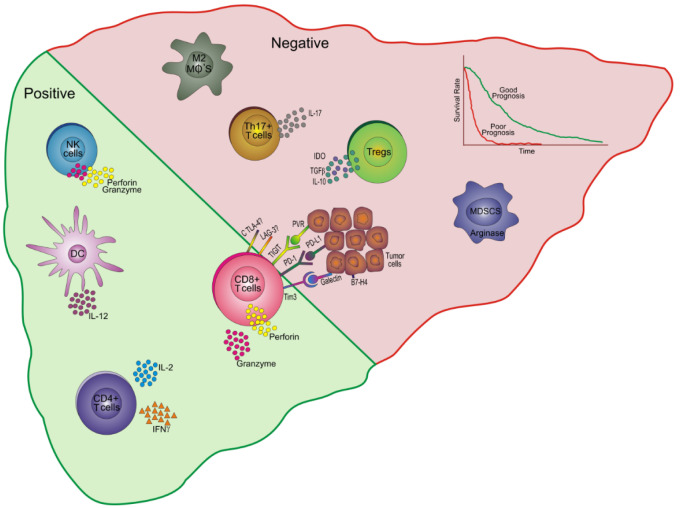
Prognostic relevance of immune cells and their associated molecules in HCC. An increased percentage of NK cells producing cytotoxic molecules like perforin and granzyme in the tumor niche are associated with good prognosis. DCs secreting IL-12 and CD4+ T cells producing cytokines, IL-2 and IFNγ are also associated with good prognosis and a favorable outcome. Higher frequencies of CD8+ T cells secreting perforin and granzyme are linked with favorable prognosis. These cells also express certain inhibitory receptors such as Tim 3, PD-1, TIGIT, CTLA-4, LAG-3 and that interact with their ligands galectin, PD-L1, PVR, CD80/CD86 and some yet unknown ligands on tumor cells leading to poor patient survival. On the other hand, Tregs secrete IDO, TGFβ and IL-10 to suppress other lymphocyte subsets and hence associate with negative or bad prognosis. Similarly, other cells like MDSCs (produce arginase), Th17 cells (IL-17) and M2 macrophages are also associated with a bad prognosis and a poor patient outcome. NK: Natural killer; DC: Dendritic cells; IL: Interleukin; IFNγ: Interferon gamma; TIM-3: T-cell immunoglobulin and mucin domain-containing-3; PD: Programmed death; TIGIT: T cell immunoglobulin and immune receptor tyrosine-based inhibitory motif domain; LAG: lymphocyte activation gene; CTLA: Cytotoxic T lymphocyte protein; PD-L: Programmed death ligand; PVR: poliovirus receptor; IDO: indolamine oxigenase; TGFβ: Transforming growth factor beta; Tregs: regulatory T cells; Th: T helper; MDSCs: Myeloid derived suppressor cells; Mφ: macrophage

A cascade of events occurs involving sequential activation of all components of immune system as a response to tumor and in turn there is subversion of immune response by the tumor for its own perpetuation. Therefore, the immune landscape in tumor environment undergoes dynamic changes that governs the course of disease and tumor metastasis. The presence of a particular type of immune cell, its localization and its functionality whether it is procancerous or cancer suppressive will have a great impact on defining prognosis. Figure 1(Fig. 1) depicts the prognostic relevance of various immune cells and their associated molecules in tumor environment of HCC.

### Natural Killer cells 

Natural killer cells (NK) cells are considered to be one of the most important players in the early immune response against a tumor. The cytotoxic effect of NK cells depends on the balance between activating and inhibitory receptors on their surface. NK cells recognize cells expressing MHC Class I, as self and do not act against them. However, tumor cells often lose their MHC I making them susceptible to the NK mediated cytotoxicity. They form immunological synapse with target cells and release cytolytic granules like perforin and granzyme to induce cell lysis. Cancers too have devised ways to rescue NK cell responses as they do for other immune cells. For example, the repertoire of activating vs inhibitory receptors gets altered leading to impaired functioning of NK cells. Therefore, strategies are being employed to potentiate the NK cell activity (Hu et al., 2019[27]). The frequency of NK cells is shown to be decreased significantly during HCC with impaired cytotoxic ability, both in circulation as well as in tumor (Cai et al., 2008[8]; Fathy et al., 2009[19]). Initially, NK cells get activated, however, during the course of carcinogenesis, they become exhausted. Suppressor cells and other factors released in tumor microenvironment interact with NK cells and directly inhibit their cytotoxicity (Liu et al., 2018[41]). The number of infiltrating NK cells correlate positively with patient survival and disease outcome (Chew et al., 2012[13], 2010[14]). Along with NK cells, there is decreased abundance of NKT cells as a component of TILs in HCC patients (Kawarabayashi et al., 2000[34]) although a similar trend in peripheral blood has not been observed (Li et al., 2017[40]). Therapeutic possibility of FoxP3 expressing NKT cells, having suppressive functions as conventional regulatory T cells and found among TILs of HCC patients, is also indicated. One study has examined the expression of TRAV10 gene as an indirect measure of NKT cells, showing their positive role in HCC prognosis (Xiao et al., 2013[72]). Although only limited studies available on NK cell subset, yet its role as a prognostic marker seems promising and needs further evaluation.

### Dendritic cells

Dendritic cells (DCs) are potent antigen presenting cells (APCs) that are capable of inducing primary immune responses and immunological memory after a pathogenic exposure. HCC patients have lesser number of circulating DCs with compromised functions such as reduced IL-12 production and T cell stimulation which may contribute to tumor progression (Kakumu et al., 2000[32]). Since DCs play pivotal role in linking innate and adaptive immunity, in most of the cancers, tumor infiltrating DCs have shown positive correlation with clinicopathological features and have promise as prognostic markers. The utility of DCs as prognostic markers in HCC was suggested as early as in 1994 where S-100 protein positive dendritic cells represented useful marker for tumor growth and prognosis (Ido et al., 1994[30]). Tumor infiltrating DCs together with lymphocytes represent as an independent prognostic factor (Yin et al., 2003[78]). In another study, it has been reported that the frequency of intratumoral DCs is speculated to serve as a predictive index for HCC recurrence and metastasis, because the number of DCs in tumor have significant correlation with frequency of memory T cells, cytotoxic T lymphocytes (CTLs) and hence high tumor-free survival in these patients (Cai et al., 2006[9]). Plasmacytoid dendritic cells (pDCs) are a subset of DCs that play important role in anti-viral immunity by secreting type I interferon (IFN). Recently, their role in cancer is getting recognized by the fact that their number in breast cancer is associated with cancer metastasis and recurrence and induce an overall immune tolerance (Treilleux et al., 2004[63]). In a recent study, pDCs have been shown to be associated with poor outcomes in patients undergoing curative resection for HCC (Zhou et al., 2019[87]). This was attributed to their capacity to induce the differentiation and expansion of Treg cells in periphery as well as in tumor region. It was proposed that these pDCs may be secreting immunosuppressive factors such as indolamine oxigenase-1 (IDO-1) or they may also promote the differentiation of Th17 cells which may contribute to a tolerogenic state and promote tumor evasion of the immune system.

### T cells

The immune cells play an important role in tumor microenvironment either as defense mechanism or the tumor turns conditions towards its own survival (Schreiber et al., 2011[56]). The CD4+ T-cells or helper T cells play a key role in antigen-specific anti-tumor responses. Tumor antigens are recognized and processed by APCs which present these antigens to CD4+ T cells in the lymph nodes. The CD4+ T cells interact with APCs through CD40-CD40L interaction and the IL-12 produced by APCs further promote the differentiation of CD4+ T cells into interferon gamma (IFN-γ)-producing type 1 T helper (Th1) cells. The CD8+ T cells on the other hand recognize processed antigens in association with MHC class I molecules displayed on DCs and differentiate into cytotoxic T lymphocytes (CTLs) that in turn produce perforin and granzyme, that aid in killing of tumor cells. In the majority of cancers, increased number of Th1 cells producing IFN-γ correlate with good prognosis. As expected, Th1 cytokines have a strong correlation with better survival of HCC patients and a Th2 cytokine repertoire with poor survival and metastatic recurrence of cancer (Budhu et al., 2006[7]). The presence of T cells in immune cell infiltrates of HCC correlate with improved survival of patients (Sun et al., 2015[60]; Garnelo et al., 2017[24]; Yao et al., 2017[76]). Despite this, there are defects at the levels of APCs with subsequent alterations in T cell activation and polarization, that ultimately aid in tumor evasion and factors released in the tissue environment also promote tumor growth.

The role of CD4+ T cells having cytotoxic effects independent of other T cells on tumor is now becoming increasingly clear in many cancers including liver cancer. An increased percentage of these cytotoxic CD4+ T cells has been shown to be associated with a strong prognosis in terms of both disease-free survival (DSF) and overall survival (OS) (Fu et al., 2013[20]). Not only number but also functional activity of these cells were independent factors for both DFS and OS. Any functional impairment would thus affect the disease progression. In this regard, regulatory T cells can potentially inhibit the release of cytolytic molecules from these cells and also can suppress generation of these cells in a dose- and contact-dependent mechanism. 

The CD8+ T cells comprise an important subset of T cells with antitumor activity mediated by release of cytotoxic molecules and hence its role in determining clinical outcomes is evident in many cancers. However, in HCC, there are contrasting reports regarding their correlation with prognosis, with majority of reports revealing its prognostic benefit (Gabrielson et al., 2016[21]; Sideras et al., 2017[59]; Ye et al., 2019[77]), while few reporting otherwise (Chen et al., 2012[12]; Ramzan et al., 2016[52]). The reasons for this discrepancy could be assessment of both number and function of CTLs in former studies, while just evaluation of frequency in latter reports. Another probable reason could be that mere presence of increased number of non-functional CTLs might actually be making tumors anergic to host defense mechanisms and refractory to the apoptotic pathways (Oudejans et al., 2005[49]). The activated state of CTLs in addition to numerical defects in tumor microenvironment should be also taken into account while considering them as prognostic markers. A recent meta-analysis incorporating the results of all these studies concluded that higher CD8+ T cell numbers were associated with high OS and DFS in patients with HCC in both intratumoral and metatumoral regions (Xu et al., 2019[73]). 

## Immune Cells Associated with Poor Prognosis in HCC

### Regulatory T cells and other suppressive cells

To counterbalance the activity of T cells, cancers have devised a suppressive array of immune cells to evade the host immune response. This includes accumulation of regulatory T cells (Tregs) and other cell populations such as myeloid derived suppressor cells (MDSCs) and tumor associated macrophages (Unitt et al., 2005[67]; Hoechst et al., 2008[26]; Shirabe et al., 2012[57]). The Tregs, characterized by expression of Fork head box protein 3 (FoxP3) transcription factor, help in maintaining immune homeostasis as a regulator mechanism in many conditions including cancer. Treg cells can suppress antitumor immunity through upregulation of cytotoxic T lymphocyte antigen 4 (CTLA-4) and suppress APCs that would further prevent activation of T cells through IL-2 depletion and release of suppressive factors such as TGF-β in the tissue environment (Togashi et al., 2019[62]). Besides this, tumor-infiltrating Treg cells also express high levels of various inhibitory molecules such as LAG3, TIM3 and GITR that confer a strong immunosuppressive activity on effector cells. More recently, Tregs have been shown to recognize self-antigens, and hence suppress the activity of self-antigen reactive CD8+ T cells. On the other hand, neo-antigen specific or non-self-specific CTLs are resistant to such immunosuppression. This observation leads to the speculation that check-point therapies would be more successful in cancers expressing high frequency of neoantigens as compared to those with low mutation rate. In such cases, targeted therapies that reduce frequency and function of Tregs would be more appropriate. As in case of any other solid tumors, an increased frequency of Tregs was consistently observed in peripheral blood and tumor tissues of HCC and predicted poor survival in these patients (Ormandy et al., 2005[47]; Yang et al., 2006[75]; Gao et al., 2007[22]). In addition, high level of TGF-β production was observed in tumor areas having high expression of FoxP3 in HCC. Therefore, tumor itself seems to secrete TGF-β, which is responsible for inducing large numbers of Tregs that are linked with poor prognosis and poor survival of HCC patients (Wang et al., 2016[68]; Yu et al., 2020[79]). While an increased ratio of CD8+ T cells to Tregs had a good prognostic ability, yet surprisingly frequency of CD8+ T cells alone did not predict the outcome (Huang et al., 2012[29]), which indicates that the fine balance between Tregs and CTLs when disrupted leads to HCC progression dominated by an increased frequency of Tregs over CD8+ T cells. 

Several independent studies have reported that increased percentage of IL-17 producing cells correlated with poor survival and tumor progression in HCC patients (Zhang et al., 2009[82]; Gu et al., 2011[25]). Similarly, a high density of Th17 cells in the tumoral areas enhance tumor growth and metastasis through upregulation of matrix metalloproteinases via activation of NF-κB (Huang et al., 2014[28]).

Myeloid-derived suppressor cells (MDSCs) are immature myeloid cells that under normal conditions differentiate into macrophages, dendritic cells or granulocytes. However, in pathological conditions, their normal differentiation is perturbed leading to their accumulation and aberrant expansion. Although MDSCs infiltrate the pathological sites in a normal physiological response to inflammation, their intrinsic suppressive property could be overpowered by excessive local inflammatory mediators causing further exacerbation of the disease. Tumor cells use MDSCs as immune escape mechanism by inducing CD8+ T cell tolerance, by expressing several immunosuppressive factors such as arginase, reactive oxygen and nitrogen species, several cytokines like IL-10 and TGF-β that inhibit T cell activation, differentiation and proliferation and also induce Tregs. Also, through engagement of several inhibiting receptors and interaction of these receptors with their cognate ligands on T cells lead to either T cell exhaustion or increased apoptosis (Nagaraj and Gabrilovich, 2008[46]). These cells have gained tremendous importance in recent years in tumor pathogenesis and metastasis, as they limit the effects of cancer immunotherapy (Nagaraj and Gabrilovich, 2007[45]). Based on their role as negative immune regulators, MDSCs are expected to be associated with an unfavorable prognosis during HCC. Several studies have reported increased percentage of MDSCs in HCC patients as compared to chronic liver disease and healthy controls, thereby suggesting their contribution towards progression from chronic hepatitis to HCC (Arihara et al., 2013[2]; Andrews et al., 2019[1]). Few meta-analyses have summarized the findings of all studies and concluded that higher MDSC levels significantly correlate with short OS and can be used as biomarker to evaluate prognosis in clinical practice (Zhang et al., 2016[83], 2019[85]).

Tumor cells also induce metabolic reprogramming in macrophages and induce infiltration of tumor associated macrophages (TAMs) or M2 macrophages for their own survival. This phenotype that leads to tumor progression is associated with large amounts of proangiogenic factors such as VEGF, IL-10, TGF-β, PGE2 and also recruits a greater number of Tregs to tumor areas. Majority of TAMs are derived from monocytes that undergo substantial alterations depending on the immunosuppressive signals from tumor microenvironment as well as tissue-specific signals (Ostuni et al., 2015[48]). Macrophages that express CD163 and CD204 are known to be associated with a M2 phenotype and correlated with a negative outcome in HCC patients (Dong et al., 2016[17]; Li et al., 2017[39]). On the other hand, macrophages expressing CD169 or M1 macrophages correlate with high numbers of infiltrating CTLs and associate with positive prognosis in HCC patients (Atanasov et al., 2019[4]). Hence, novel therapies that could skew the macrophages towards a M1 type would be worth investigating.

### Immune check points as potential prognostic markers

The mechanisms of immune escape of tumors are although less understood, yet, it is generally accepted that it plays an essential role in carcinogenesis, progression and metastasis of almost all types of cancer. With recognition of immune checkpoint (IC) molecules and their role during immune homeostasis, they have become good therapeutic targets with promising results (Perez-Gracia et al., 2014[51]). Clinical trials with antibodies against PD-1 (nivolumab and pembrolizumab) and PD-L1 (atezolizumab) are under way in HCC patients (Pardoll, 2012[50]; Kudo, 2017[35]). It becomes relevant to explore their role as robust prognostic markers and many studies on HCC have addressed this issue. An aberrant expression of PD-L1 has been correlated with cancer progression and its blockade enhanced the anti-tumor response. High expression of PD-L1 can turn off the activated state of anti-tumor cells and may skew intrinsic anti-oncogenic signaling pathways of the infiltrating immune cells. In these lines, PD-L1 expression has been associated with a poor overall survival rate as reported by several studies (Gao et al., 2009[23]; Umemoto et al., 2015[66]). On the contrary, a positive effect of its expression on disease outcome has also been reported in a couple of studies (Chen et al., 2016[11]; Sideras et al., 2017[59]). A meta-analysis based on 17 studies with 2979 patients, has reported no significant prognostic role of PD-L1 in HCC after curative hepatectomy which essentially warrants further investigation of its role as a predictive marker (Andrews et al., 2019[1]). Interestingly, in this study, differences were found with respect to population cohorts, with correlation of high PD-L1 with poor survival in Asian studies and better disease outcome in non-Asian cohorts. There are several other factors which must be kept in mind before marking PD-L1 as prognostic, like its expression on tumor cells or immune cells or overall expression on intra-tumoral area, the analytical method to determine its expression, staining pattern, cut-off values and most importantly the baseline characteristics of patients.

There are multiple immune check points besides PD-1/PD-L1 whose distribution in the HCC patients need to be assessed owing to dearth of knowledge in this area. Since a significant proportion of patients do not respond to monotherapy targeting immune checkpoints, combinatorial strategies employing additional molecules will be more useful in predicting disease outcome. Another negative regulator of T cell response, V-set domain-containing T-cell activation inhibitor 1 (VTCN1 or B7-H4) which is a ligand for B and T cell lymphocyte attenuator (BTLA) was found to be significantly upregulated in HCC patients, although normal cells do not express this molecule (Kang et al., 2017[33]). The signaling mechanism regulating B7-H4 remains largely unknown and guided by the complexity of tumor environment and unlike other B7 family molecules, its expression is not tightly regulated (Sica et al., 2003[58]). Since binding of B7-H4 with its ligand inhibits T cell responses, its expression on tumor tissue and in circulation of HCC patients predicted poor overall survival and remission-free survival (Zhang et al., 2015[80][84]). These studies emphasize its utility as a novel biomarker and warrants future studies as a potential therapeutic target particularly in patients who do not respond to current check point blockers and for predicting HCC prognosis and recurrence.

T-cell immunoglobulin and mucin domain-containing-3 (TIM-3) is an immune checkpoint molecule that binds to its ligand Galectin-9 and curtail T-cell mediated immune responses (Tang et al., 2019[61]). Tim-3 receptor activates NF-κB signaling cascade that leads to increased secretion of IL-6 that helps in the proliferation of tumor cells (Zhang et al., 2018[81]). Increased number of Tim3+ TILs were observed in HCC patients, co-localized with galectin-9+ cells. Their interaction leads to immune-dysfunction manifested by decreased T cell proliferation and effector functions of CD4+ T cells. Blocking this pathway reprograms the cell's senescence and functionality as well (Zhou et al., 2017[86]). Expression of Tim-3 was also negatively associated with disease outcome in terms of shorter survival of patients, metastasis, and tumor-recurrence (Li et al., 2012[38]). Not only the surface of tumors, soluble Tim-3 levels were also found to be associated with poor survival of HCC patients (Li et al., 2018[36]). Together with PD-1, Tim-3 expressing T cells displayed lowest level of granzyme B, IFN-γ, and TNF-α, in HCC microenvironment suggesting an important role in T-cell suppression. Blocking individual inhibitory receptor had variable response as per the expression level of each of its receptors, however, combinatorial blockade of PD-L1 with TIM3, LAG3, or CTLA4 had positive impact on most of the patients and suggests about additive benefits over monotherapy. The long term effects of such blockade therapy and other factors like host genetic background and also the effect of environmental factors remains to be seen. The application of such therapies should proceed with caution since normal homeostasis particularly at peripheral sites is disrupted following check point blockade. Therefore, it is suggested to explore novel receptors and targeting them with tumor-restricted approaches such that a potent immune response is elicited in tumor while hyper activation of the immune response against self-antigens in periphery is circumvented (Schnell et al., 2020[55]).

T cell immunoglobulin and immune receptor tyrosine-based inhibitory motif domain (TIGIT), a member of the immunoglobulin superfamily, whose ligand is poliovirus receptor, CD155. It is predominantly expressed on activated T cells with a cell-intrinsic inhibitory effect and on NK cells where it inhibits cytotoxicity (Andrews et al., 2019[1]). An increased expression of TIGIT and its ligand were observed in HCC patients, suggesting its role in the pathogenesis of HCC (Duan et al., 2019[18]). Although its role as a prognostic marker is indicated, larger studies need to be undertaken to validate this statement. In a recent study from 2019, combined expression of PD-1 and TIGIT on circulating CD8+ T cells was associated with a poor HBV-HCC prognosis (Liu et al., 2019[42]). Interestingly, TIGIT has been proposed to play distinct roles during tumor initiation and progression, hence the use of check point inhibitors against TIGIT would result in different outcomes depending on the stage of tumor (Zong et al., 2019[88]). Before HBV/HCV mediated tumor initiation, TIGIT expression helps in maintaining the immune tolerance in hepatic tissue so that there is minimal immune mediated pathology, however, sustained expression of TIGIT promotes the progression of tumor by diminishing anti-tumor immunity.

In recent years, another inhibitory molecule belonging to Immunoglobulin superfamily, the lymphocyte activation gene-3 (LAG-3) located very closely to CD4, has gained considerable attention and its role needs to be extensively explored. It is expressed on CD4+ T cells, CD8+ T cells, Tregs and NK cells and it interacts with its ligand, MHC Class II mainly expressed on APCs which negatively regulates T cell proliferation and activation and also down-regulated DC functions (Workman et al., 2002[70]; Workman & Vignali, 2003[71]). LAG-3 is a unique checkpoint molecule that differs structurally as well as functionally from other immune check point molecules. Structurally, its cytoplasmic tail has three conserved domains and it competes with CD4 for binding to same MHC-II molecule hindering the TCR signaling and hence causing impaired T cell proliferation (Long et al., 2018[43]). LAG-3 associates with PD-1 in attenuating immune response and dual blockade with anti-LAG-3 and anti-PD-1 has shown efficacy in melanoma patients and benefitted patients who have shown no response to anti-PD-1/PD-L1 therapy (Ascierto et al., 2017[3]). However, the mechanism behind this complicated interaction with PD-1 and LAG-3 is still obscure. LAG-3 expression was significantly upregulated in tumor infiltrating CD8+ T cells and correlate significantly with CD8+ T cell dysfunction among HCC patients (Li et al., 2013[37]). There are still unanswered questions regarding its role as prognostic marker in many types of carcinomas.

Cytotoxic T lymphocyte protein 4 (CTLA-4) share its ligands, CD80 and CD86 with CD28 and hence antagonize with the costimulatory signals provided by CD28 binding for T cell activation. CTLA-4 shares functional similarity with LAG-3 in inhibiting TCR signaling pathway and acts at an early stage of T-cell activation. Cells in resting state express CTLA-4 intracellularly and it is only after T cell activation following CD28/B7 binding leads to its translocation to the surface. It binds to B7 with a higher affinity and prevents further activation of T cells and cell cycle progression. Since CTLA-4 is also expressed on regulatory T cells where it helps in their T cell suppressive functions, therefore, blocking it would reduce Treg-mediated suppression and proliferation of effector T cells (Buchbinder and Desai, 2016[6]). The role of CTLA-4 as predictive biomarker has not been much evaluated and in one study it showed no significant association with the prognosis and clinicopathologic factors since only non-specific staining of CTLA-4 was observed in tissue sections (Chang et al., 2017[10]). 

### Methods to monitor prognostic markers

The intensive characterization of tumor microenvironment with respect to immune cell evaluation and monitoring will help to predict disease outcome in patients with cancer. The techniques used also impact significantly the prospective utility of a marker as a predictive factor and help to innovate strategies for better management of disease course. The characteristics of a robust biomarker defines early identification of patients who are at risk, relative ease of measuring that biomarker, possibly in a sample that is easier to obtain with nominal cost to the patient (Tripepi et al., 2010[64]). Many potential biomarkers do not receive regulatory approval despite preliminary clinical evaluations. This is because such studies are generally performed on small populations and hence lack power and not validated properly. It is only after proper validation that a biomarker can be utilized by clinicians on vast majority of patients which is measured by its accuracy and generalizability. Accuracy can be defined as a correct prediction about an outcome through measurement of a biomarker or prognostic marker. Generalizability is the ability of a biomarker to predict a consequence in a sample population and generalize this to whole population cohort other than in which the biomarker has gone through validation. While defining any marker as prognostic, the assessment strategies should be kept consistent. For example, the study should begin during a particular stage of disease in a defined population and patients should be followed for sufficient time with similar follow up periods. Besides death and recovery, other factors such as age, tumor size, pain, disability and likelihood of benefit from treatment should also be considered.

In general, Immunohistochemistry (IHC) remains the workhorse for the majority of pathologists for routine evaluation of disease status and to determine the prognostic ability of markers in cancer tissues. Advancements have led to development of human protein atlas program with expansion of a plethora of antibodies specific for most proteins expressed in normal tissues or cancer tissues (Uhlen et al., 2005[65]). Other techniques such as flow cytometry and DNA microarrays have revolutionized the field of medical and translational research, however, not routinely employed clinically in the field of oncology owing to high cost and instrument complexity, besides the technical expertise needed to use these technologies. Moreover, these newer technologies create a very large amount of data that needs to be analyzed using sophisticated statistical tools to select a few relevant biomarkers. 

To evaluate the prognostic relevance of a biomarker, a useful method is receiver operator characteristic (ROC) curve analysis. Here, assessment between a binary marker and risk of an event is depicted in the survival curve. A positive marker (high risk group) is plotted against a negative marker (low risk group) and separation between the survival curves represents the probability that a longer lifetime a patient has in the low risk group than a patient in the high risk group (Combescure et al., 2014[16]). One limitation of ROC curve analysis is that the cut off drawn is dichotomous based on alive or dead regardless of follow-up time. Multiple regression models are used for analysis of a prognostic marker, most common being the Cox model. This statistical technique evaluates correlation between a patient's survival and simultaneously effect of several covariates or predictor variables. It allows for the estimation of risk of death for an individual for a given prognostic variable. This model yields a formula in the form of hazard ratios to assess the risk from several predictor variables. A positive regression coefficient related to the hazards from this equation corresponds to a bad prognosis, while a negative coefficient means, a protective effect or better prognosis (Collett, 2014[15]; Machin et al., 2006[44]).

### Future prospects

Recent insights into the molecular hepatocarcinogenesis have highlighted the role of immune landscape in shaping the overall anti-tumor response and therapeutic outcomes. With the advent of newer immune check points and their potential role as prognostic markers would improve the therapeutic outcomes for HCC patients. These molecules should be assessed in larger cohorts of patients with proper validation in control trials. The prognosis research standards should be improved for translating clinical research to clinical practice. In the era of genomics, future developments in the discovery of novel gene expression signatures of tumor infiltrating immune cells will significantly impact the prognosis research. The identification of new prognostic factors may also broaden categorization of patients suitable for a particular treatment. Predicting risk and outcome is usually insufficient on the basis of a single prognostic factor, hence, in future multiple prognostic factors should be evaluated using advanced prognostication models and algorithms.

## Summary and Conclusions

The role of immune cells in cancer pathogenesis is now well accepted and recent studies have delineated pathways and mechanisms governing cellular interactions. The cancer tissue microenvironment is suppressive in nature because cancer cell continually evolves new mechanisms of immune evasion. Both kinds of tumor infiltrating cells, anti-tumor and pro-tumorous that either aid in tumor suppression or foster tumor progression respectively are present in tumor areas. The dynamic interplay among all cells of the immune system and tumor environment modulate the fate of tumor. All these TILs and their secreted factors have a great impact on tumor prognosis and response to therapy. Tumor infiltration by immune cells such as T cells, DCs, NK cells, NKT cells is linked with improved prognosis and favorable outcome. An inhibition of these cells by the virtue of Tregs or MDSCs on the other hand is associated with poor clinical outcome. Not only their frequencies, the immune cells gradually lose their functionality owing to increased expression of immune check points. Overexpression of these negative receptors provide inhibitory signals to T cells and they are also associated with poor prognosis, although research is underway in evaluating these markers as prognostic factors in larger cohorts of patients. Not only this, research on targeting these factors is actively ongoing and among the thrust areas for the development of novel therapeutics. Strategies to deplete the immunosuppressive cell populations also hold promise to confer benefits as immunotherapy against HCC.

## Conflict of interest

The authors declare that they have no conflict of interest.

## Acknowledgements

Authors sincerely acknowledge the help from Mr. Dharamjit Singh, Department of Experimental and Medical Biotechnology, PGIMER Chandigarh, India for graphical designing of the figure.

## Figures and Tables

**Table 1:**
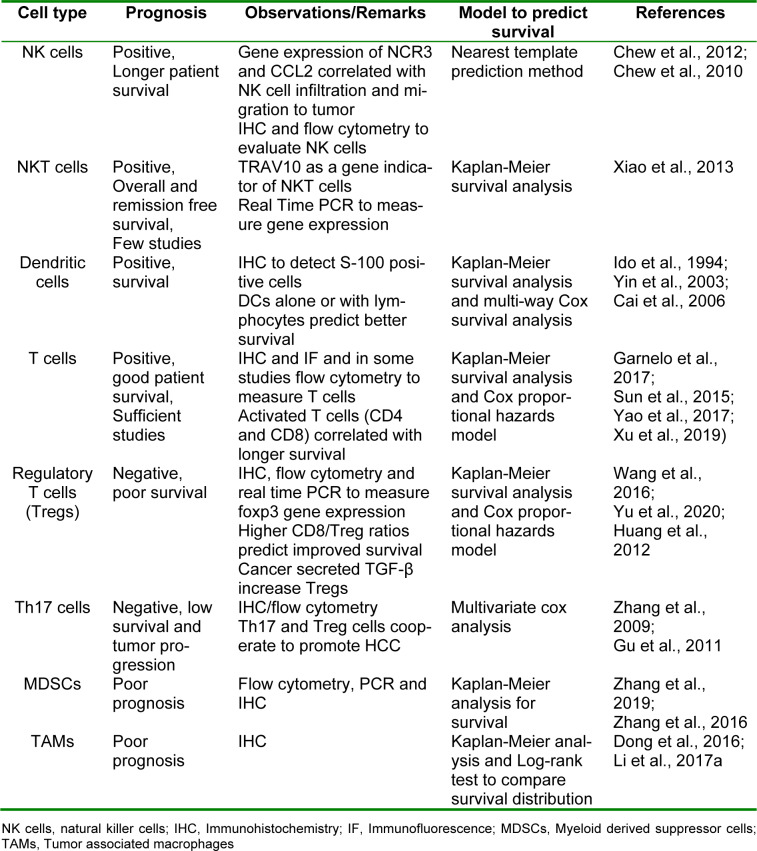
Summary of studies on the prognostic importance of various immune cells in HCC
